# Image Similarity Quantum Algorithm and Its Application in Image Retrieval Systems

**DOI:** 10.3390/e27020137

**Published:** 2025-01-27

**Authors:** Qingchuan Yang, Xianing Feng, Lianfu Wei

**Affiliations:** 1 Information Quantum Technology Laboratory, International Cooperation Research Center of China Communication and Sensor Networks for Modern Transportation, School of Information Science and Technology, Southwest Jiaotong University, Chengdu 610031, China; yangqingchuan@my.swjtu.edu.cn (Q.Y.); fengxn5@swjtu.edu.cn (X.F.); 2 Sichuan Zhitu Linghai Intelligent Technology Co., Ltd., Chengdu 610213, China

**Keywords:** image similarity, AE-QIP, ST-QIP, image retrieval, algorithm performance

## Abstract

The measurement of image similarity represents a fundamental task within the domain of image processing, enabling the application of sophisticated computational techniques to ascertain the degree of similarity between two images. To enhance the performance of these similarity measurement algorithms, the academic community has investigated a range of quantum algorithms. Notably, the swap test-based quantum inner product algorithm (ST-QIP) has emerged as a pivotal method for computing image similarity. However, the inherent destructive nature of the swap test necessitates multiple quantum state evolutions and measurements, which leads to consumption of quantum resources and prolonged computational time, ultimately constraining its practical applicability. To address these limitations, this study introduces an advanced quantum inner product algorithm based on amplitude estimation (AE-QIP) designed to compute image similarity. This innovative approach circumvents the repetitive measurement processes associated with the swap test, thereby optimizing the utilization of quantum resources and substantially enhancing the algorithm’s performance. We conducted experiments using a quantum simulator to implement the AE-QIP algorithm and evaluate its effectiveness in the image retrieval tasks. It is found that the AE-QIP algorithm achieves comparable precision to the ST-QIP algorithm while exhibiting significant reductions in qubit consumption and average processing time. Additionally, our findings suggest that increasing the number of ancillary qubits can further enhance the accuracy of the AE-QIP algorithm. Overall, within the acceptable error thresholds, the AE-QIP algorithm exhibits enhanced efficiency relative to the ST-QIP algorithm. However, significant further research is needed to address the challenges involved in optimizing the performance of quantum retrieval systems as a whole.

## 1. Introduction

With the sustained advancements and applications of digital image processing technology, image similarity algorithms have emerged as critical components in the domains of computer vision and image retrieval. These algorithms are designed to assess the similarity between images by calculating quantitative measures of their likeness. Prominent methodologies include the histogram approach  [1,2], the cosine similarity method  [3,4], jaccard similarity  [5], and hash value techniques  [6]. Among these techniques, cosine similarity is particularly prevalent in evaluating the similarity of both textual and visual data.

In order to apply cosine similarity to image comparisons, it is essential to convert the images into vector representations. The subsequent computation of cosine similarity involves evaluating the angle between these vectors, which can be expressed through the inner product of the $L_{2}$ normalized vectors  [7]. Notably, the computational complexity of this inner product operation is positively correlated with the dimensionality of the vectors; as the feature vector dimensions increase, the computation time correspondingly escalates. Moreover, the complexity of image retrieval intensifies as the volume of images involved in comparison increases.

In response to these challenges, an increasing number of researchers have proposed algorithms aimed at accelerating image retrieval processes. This often entails the application of unsupervised learning clustering techniques  [8] to effectively partition the database into K sub-datasets based on image similarity. By confining comparisons to the most similar sub-datasets, rather than evaluating the entire database, these methods significantly enhance retrieval efficiency.

While clustering algorithms undoubtedly improve retrieval efficiency, they primarily serve to reduce the volume of data that requires comparison, without fundamentally altering the intrinsic complexity of the image similarity algorithms themselves. Therefore, this paper concentrates on advancing various aspects of the image similarity algorithm. A review of the existing literature reveals that numerous studies have successfully applied image similarity algorithms in practical applications such as face recognition  [9], object tracking  [10], and person re-identification  [11]. However, there remains a notable dearth of research addressing the application of quantum algorithms to image similarity, indicating an area ripe for further exploration.

As quantum computing technology continues to advance, researchers have developed a range of quantum algorithms that leverage the properties of quantum coherence and entanglement. The implementation of classical algorithms has been accelerated, as noted in several studies  [12,13,14]. Notably, some quantum algorithms can achieve exponential speedup; for instance, quantum amplitude estimation holds the potential to provide a quadratic speedup in simulating random processes, offering performance improvements over classical Monte Carlo methods  [15]. Additionally, the quantum support vector machine  [16,17,18] and the enhanced HHL algorithm  [19] further exemplify this progress.

Recognizing the significant role of quantum algorithms in quantum information processing, researchers have recently introduced a series of algorithms based on the swap test  [20,21]. These utilize quantum parallel computing to implement quantum state inner product calculations. Since cosine similarity can be derived from inner products, this approach can facilitate image retrieval. However, the quantum inner product algorithm based on the swap test (ST-QIP) is a destructive test, requiring multiple measurements for accurate results  [22], and presents a high algorithmic complexity.

In contrast, the quantum amplitude estimation algorithm  [15,23], as a fundamental application-oriented quantum algorithm applicable to Monte Carlo integration and machine learning tasks, has garnered significant attention. Building upon this quantum amplitude estimation algorithm, we present the quantum inner product algorithm for amplitude estimation (AE-QIP) in this paper, which applies a more efficient method for calculating image similarity. This algorithm necessitates only a single measurement to derive results, potentially making it more advantageous than the ST-QIP approach.

In this paper, we introduce a novel methodology for image similarity comparison that leverages classical neural networks to derive feature vectors from images. These feature vectors are subsequently transformed into normalized feature vectors using the $L_{2}$ norm, which are then encoded as quantum states within a Hilbert space framework. To efficiently perform the inner product calculations of these quantum feature vectors, we employ the AE-QIP algorithm. Notably, this approach facilitates the computation of cosine similarity through a single measurement, marking a significant advancement in the efficiency of image similarity comparisons. This methodology not only enhances computational performance but also opens new avenues for integrating quantum computing techniques into image processing applications.

Notably, the AE-QIP algorithm achieves high accuracy levels through the augmentation of auxiliary qubits, eschewing the need for an increased number of repeated measurements, thus offering the potential for quantum speedup. Our complexity analysis indicates that the AE-QIP algorithm markedly reduces both time and space complexity relative to the ST-QIP algorithm while remaining within the bounds of practical error tolerance.

The structure of this paper is organized as follows: In Section 2, we establish that computing cosine similarity is mathematically analogous to calculating the inner product of normalized feature vectors. This transformation simplifies the image similarity algorithm and lays the theoretical groundwork for both the AE-QIP and ST-QIP algorithms. Section 3 provides a theoretical analysis of the algorithmic complexity, demonstrating that the AE-QIP algorithm possesses lower complexity than its ST-QIP counterpart within defined error margins. In Section 4, we empirically validate the efficacy of the AE-QIP algorithm using the Qiskit simulator and analyze how the number of auxiliary qubits influences the algorithm’s performance, contrasting it with the ST-QIP algorithm. Finally, Section 5 summarizes the findings and limitations of this research while proposing directions for future investigation.

## 2. Fundamentals of Quantum Algorithms for Image Similarity

The image retrieval algorithm focuses on assessing the similarity between a query image and comparison images stored within a database. This is commonly achieved by computing the cosine similarity of the image feature vectors, which can be simplified to the computation of the inner product of two normalized feature vectors. To exploit the advantages of quantum parallel computation, this study proposes a quantum algorithm for calculating image similarity. Initially, we theoretically elucidate the relationship between quantum inner product algorithms and their classical counterparts. Subsequently, the quantum inner product algorithm based on amplitude estimation is derived, and the corresponding quantum circuit is constructed. In addition, for comparative analysis, the ST-QIP algorithm is derived and its quantum circuit is established.

### 2.1. Establishing the Connection Between Classical Inner Products and Quantum State Inner Products

Mathematically, the similarity between two vectors of identical dimensions can be measured through cosine similarity. For instance, consider two *N*-dimensional vectors, $\vec{A}=\left(x_{1},x_{2},\ldots ,x_{N}\right)$ and $\vec{B}=\left(y_{1},y_{2},\ldots ,y_{N}\right)$. The cosine similarity can be expressed as follows:

(1) $$\cos\left(\theta \right)=\frac{\vec{A}\cdot \vec{B}}{\left|\right|\vec{A}\left|\right|\cdot \left|\right|\vec{B}\left|\right|}=\frac{\sum_{i=1}^{N}\left(x_{i}\cdot y_{i}\right)}{\sqrt{\sum_{i=1}^{N}x_{i}^{2}}\cdot \sqrt{\sum_{i=1}^{N}y_{i}^{2}}}$$

If we consider the vectors in their $L_{2}$ normalized forms, we have the following:

(2) $${\vec{A}}^{\prime }=\frac{\vec{A}}{\left|\right|\vec{A}\left|\right|}=\left[x_{1}^{\prime },x_{2}^{\prime },\ldots ,x_{N}^{\prime }\right],{\vec{B}}^{\prime }=\frac{\vec{B}}{\left|\right|\vec{B}\left|\right|}=\left[y_{1}^{\prime },y_{2}^{\prime },\ldots ,y_{N}^{\prime }\right]$$

Consequently, the cosine similarity between the $L_{2}$ normalized vectors ${\vec{A}}^{\prime }$ and ${\vec{B}}^{\prime }$ can be expressed as their inner product:

(3) $$\cos\left(\theta \right)={\vec{A}}^{\prime }\cdot {\vec{B}}^{\prime }=\sum_{i=1}^{N}\left(x_{i}^{\prime }\cdot y_{i}^{\prime }\right),$$

The resultant values lie within the interval cos(θ)∈[−1,1]. A cosine similarity value closer to 1 indicates greater similarity between the two vectors. Thus, computing the cosine similarity of two vectors can effectively be translated into determining the inner product of their $L_{2}$ normalized equivalents. The computational complexity of the classical inner product algorithm for two *N*-dimensional vectors is O(N), indicating that the consumption of computational resources increases linearly with the vector’s dimensionality.

Fortunately, quantum superposition, a fundamental property of quantum mechanics, offers an efficient mechanism for implementing quantum vector inner products. In Hilbert space, the quantum state corresponding to *n* qubits can be represented as a $2^{n}$-dimensional vector. When the feature vector dimension is *N*, the requisite number of qubits to amplitude encode a quantum state is merely $\left\lceil \log_{2}\left(N\right)\right\rceil$. For example, any normalized two-dimensional vector can be represented as a general state of the qubit: |ψ〉=α|0〉+β|1〉, where |0〉 and |1〉 denote the basis vectors, with the condition that ${\left|\alpha \right|}^{2}+{\left|\beta \right|}^{2}=1$. The matrix forms of the basis vectors |0〉 and |1〉 can be expressed as follows: $|0\rangle ={\left[1\;0\right]}^{T}$ and $|1\rangle ={\left[0\;1\right]}^{T}$. Thus, any arbitrary *N*-dimensional normalized vector $Z={\left[a_{1},a_{2},\ldots ,a_{N}\right]}^{T}$ may be represented through a *N*-dimensional wave function in Hilbert space: $|\psi \rangle =\sum_{i=1}^{N}c_{i}|i\rangle$, where |i〉 indicates a direct product state. Conversely, quantum states in *N*-dimensional Hilbert spaces can likewise be utilized to encode a $L_{2}$ normalized *N*-dimensional vector. Consequently, the two $L_{2}$ normalized *N*-dimensional vectors ${\vec{A}}^{\prime }$ and ${\vec{B}}^{\prime }$ can be encoded as quantum states $|\varphi \rangle ={\left[x_{1}^{\prime },x_{2}^{\prime },\ldots ,x_{N}^{\prime }\right]}^{T}$ and $|\phi \rangle ={\left[y_{1}^{\prime },y_{2}^{\prime },\ldots ,y_{N}^{\prime }\right]}^{T}$, where $x_{i}^{\prime }$ and $y_{i}^{\prime }$ are arbitrary complex numbers. The inner product of the quantum states |φ〉 and |ϕ〉 is given as follows:

(4) $$\langle \varphi |\phi \rangle =\left[x_{1}^{\prime *},x_{2}^{\prime *},\ldots ,x_{N}^{\prime *}\right]{\left[y_{1}^{\prime },y_{2},\ldots ,y_{N}\right]}^{T}=\sum_{i=1}^{N}x_{i}^{\prime *}y_{i}^{\prime },$$

From Equations (3) and (4), we can establish the following relationship:

(5) $$\cos\left(\theta \right)={\vec{A}}^{\prime }\cdot {\vec{B}}^{\prime }=\langle \varphi |\phi \rangle$$

This indicates that the inner product of a $L_{2}$ normalized *N*-dimensional vector is numerically equivalent to the inner product of the corresponding quantum states, achievable through the implementation of specific quantum algorithms. Thus, a theoretical foundation is established for addressing classical inner products via quantum computational methodologies.

### 2.2. ST-QIP Algorithm

**Figure 1:**
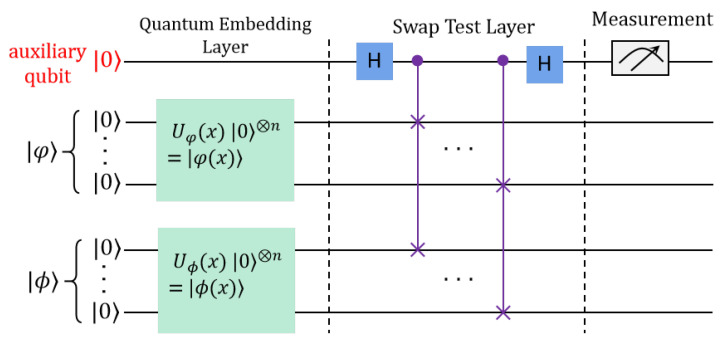
Quantum circuit implementation for the inner product between |φ〉 and |ϕ〉 using the swap test quantum algorithm.

The quantum inner product algorithm holds a critical position in the domain of quantum information processing, prompting the development of various quantum algorithms designed to efficiently compute the required quantum inner product, thereby facilitating a range of applications  [24]. The seminal work by Buhrman et al.  [25] introduced the swap test method for calculating the inner product of quantum vectors, known as the swap test quantum inner product (ST-QIP). As illustrated in Figure 1, the quantum circuit for ST-QIP comprises Hadamard (*H*) gates and controlled swap (*C*-SWAP) gates.

In this study, we consider the encoding of an *N*-dimensional vector ${\vec{A}}^{\prime }=\left(x_{1},x_{2},\ldots ,x_{N}\right)$ as a quantum state |φ〉, while another *N*-dimensional vector ${\vec{B}}^{\prime }=\left(y_{1},y_{2},\ldots ,y_{N}\right)$ is represented as the quantum state |ϕ〉. Following the quantum embedding layer, the initial quantum state ${|0\rangle }^{⊗2N}|0\rangle$ is transformed into the composite state $|\psi_{0}\rangle =|\phi \rangle |\varphi \rangle |0\rangle$.

In addition to the working qubits $\left\{q_{1},\ldots ,q_{2N}\right\}$, an auxiliary qubit $q_{0}$ is introduced. This auxiliary qubit serves a dual purpose: it not only controls the working qubits to implement the swap gate necessary for the computation of the desired inner product, but it also facilitates the readout of the computation results. Following the application of the swap test layer, the quantum state $|\psi_{0}\rangle$ is evolved according to the prescribed operations:

(6) $$|\psi_{1}\rangle =\frac{1}{2}(|\phi \rangle |\varphi \rangle +|\varphi \rangle |\phi \rangle )|0\rangle +\frac{1}{2}(|\phi \rangle |\varphi \rangle -|\varphi \rangle |\phi \rangle )|1\rangle .$$

The computation result represented by the inner product state $|\psi_{1}\rangle$ can be probabilistically obtained via projective measurement on the control qubit $q_{0}$. Given that this measurement pertains to a single qubit, the measurement operator can be articulated as ${\hat{M}}_{0}=\left|0\rangle \langle 0\right|$. The probability that the auxiliary qubit collapses to the state |0〉 following the projective measurement is expressed as follows:

(7) $$P_{0}=\frac{1}{2}\left(1+{\langle \varphi |\phi \rangle }^{2}\right).$$

This relationship elucidates that the inner product between the quantum states |φ〉 and |ϕ〉, or equivalently, the inner product between the $L_{2}$ normalized vectors ${\vec{A}}^{\prime }$ and ${\vec{B}}^{\prime }$, can be articulated as follows:

(8) $${\vec{A}}^{\prime }\cdot {\vec{B}}^{\prime }=\langle \phi |\varphi \rangle =\sqrt{2P_{0}-1}.$$

Consequently, the inner product of the vectors ${\vec{A}}^{\prime }$ and ${\vec{B}}^{\prime }$ can be computed with relative ease using this quantum algorithm. Despite the attention garnered by the swap test as a quantum inner product algorithm, it is noteworthy that the quantum state becomes maximally entangled post-measurement due to the destructive nature of the swap test  [26]. Moreover, given the non-clonability of quantum states, it is imperative to prepare O(1/ϵ) instances of the quantum states |ϕ〉 and |φ〉 to achieve the inner product with a defined error margin ϵ. This necessity culminates in a high computational complexity for the quantum inner product algorithm, necessitating substantial quantum resources.

### 2.3. Algorithm in This Paper: AE-QIP Algorithm

**Figure 2:**
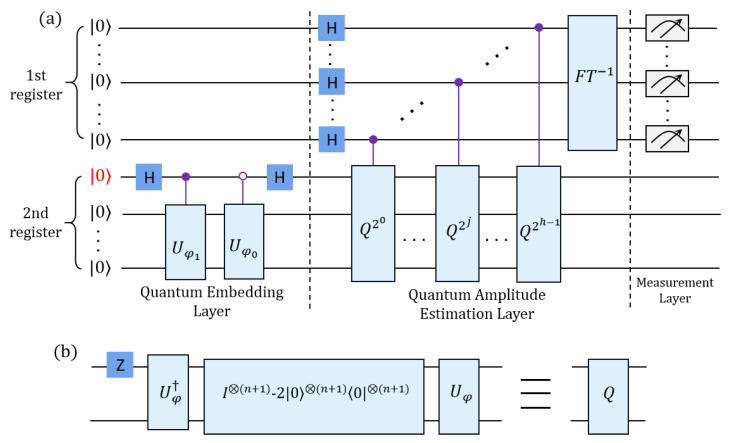
(**a**) Quantum circuits for the AE-QIP algorithm. (**b**) Details of Q operator in AE-QIP algorithm.

The quantum inner product 〈φ|ϕ〉, which quantifies the overlap between the states |φ〉 and |ϕ〉, is a fundamental concept in quantum mechanics. It plays a pivotal role in quantum information processing  [24]. For example, the absolute value of this inner product measures the distance or indistinguishability between two quantum states, while the square of this absolute value is commonly referred to as fidelity. A higher fidelity suggests that more quantum resources are necessary to determine the distance between the two states. Given that the destructive measurement associated with the swap test-based quantum inner product algorithm (ST-QIP) requires considerable quantum resources, we propose a quantum inner product algorithm that employs amplitude estimation (AE-QIP). The quantum circuit diagram for this algorithm is presented in Figure 2a.

The algorithm begins by preparing the initial quantum state ${|0\rangle }^{h+n+1}$, where the first register comprises *h* auxiliary qubits and the second register contains n+1 qubits for data encoding. The number of qubits needed to represent the amplitude of an N-dimensional feature vector is given by $n=\lceil \log_{2}^{N}\rceil$. The quantum embedding layer of the AE-QIP algorithm utilizes the unitary operator $U_{\left(\varphi \right)}$ to act on the second register, effectively encoding the classical feature vector into the corresponding quantum state such that

(9) $$U_{\left(\varphi \right)}{|0\rangle }^{⊗n}|0\rangle =1/2[(|\varphi_{0}\rangle +|\varphi_{1}\rangle )|0\rangle +(|\varphi_{0}\rangle -|\varphi_{1}\rangle )|1\rangle ]$$

Let |u〉 represent the state corresponding to the superposition $|\varphi_{0}\rangle +|\varphi_{1}\rangle$, and let |v〉 denote the state associated with the superposition $|\varphi_{0}\rangle -|\varphi_{1}\rangle$. It follows that there exists a real number θ such that

(10) $$U_{\left(\varphi \right)}{|0\rangle }^{⊗n}|0\rangle =\sin\left(\theta \right)|u\rangle |0\rangle +\cos\left(\theta \right)|v\rangle |1\rangle$$

Let $|\psi_{0}\rangle$ and $|\psi_{1}\rangle$ be defined as |u〉|0〉 and |v〉|1〉, respectively. We can express the combined state as follows:

(11) $$|\varphi \rangle =U_{\left(\varphi \right)}{|0\rangle }^{⊗n}\left|0\rangle =\sin\left(\theta \right)\right|\psi_{0}\rangle +\cos\left(\theta \right)|\psi_{1}\rangle$$

Here, $|\psi_{0}\rangle$ is referred to as the normalized bad state, while $|\psi_{1}\rangle$ is known as the good state. To estimate the probability that |φ〉 yields a good state upon measurement, we make use of the quantum amplitude estimation layer, which calculates $\langle \psi_{1}|\psi_{1}\rangle$. The probability of obtaining a good state can be enhanced through the application of the unitary operator *Q*, as illustrated in Figure 2b:

(12) $$Q=-U_{\left(\varphi \right)}S_{0}U_{\left(\varphi \right)}^{†}S_{\psi_{1}}$$

In this context, $U_{\left(\varphi \right)}^{†}$ denotes the complex conjugate transpose of the matrix $U_{\left(\varphi \right)}$, $S_{0}=I^{⊗(n+1)}-{2|0\rangle }^{⊗(n+1)}{\langle 0|}^{⊗(n+1)}$, and $S_{\psi_{1}}=I^{⊗(n+1)}-2|\psi_{1}\rangle \langle \psi_{1}|$.

In the AE-QIP algorithm, the first register contains *h* qubits initially set to ${|0\rangle }^{⊗h}$, which are then transformed into equal superposition states to store the results. This first register acts as a control gate for the successive application of the controlled *Q* operator on the second register. Prior to measurement, the inverse quantum Fourier transform ($FT^{†}$) is applied to all qubits in the first register. Afterward, amplitude estimation is used to measure the integer y∈{0,…,H−1}, which is mapped to the angle $\theta^{\prime }=\frac{y\pi }{H}$ [27], where $H=2^{h}$. Furthermore, the inner product of the quantum states is obtained as follows:

(13) $$\langle \varphi_{1}|\varphi_{0}\rangle =-\cos\left(2\theta^{\prime }\right)=-\cos\left(\frac{\pi y}{2^{h-1}}\right).$$

The algorithm encodes the quantum inner product information into the qubits of the first register using quantum amplitude transformation and is capable of calculating the inner product by measuring the first register just once. In theory, the error rate of the algorithm diminishes as the number of qubits in the first register increases. The ST-QIP algorithm requires multiple destructive exchange tests, and its accuracy is contingent upon the number of exchange tests conducted, which corresponds to the number of times the entire quantum circuit is executed. Consequently, when addressing the quantum inner product problem, the AE-QIP algorithm demonstrates superior performance advantages over the ST-QIP algorithm in theory.

**Figure 3:**
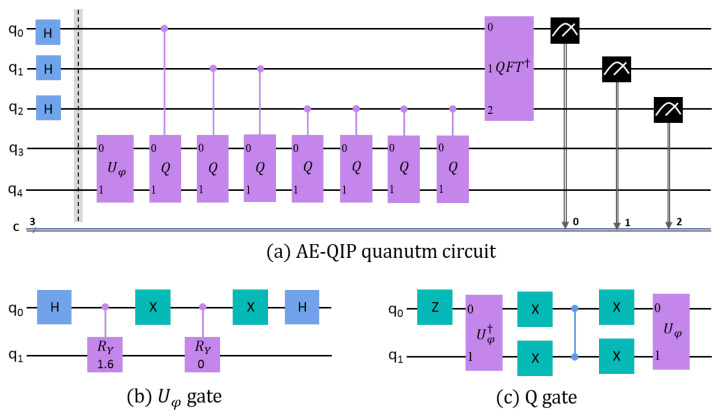
(**a**) The quantum circuit representing the AE-QIP algorithm constructed using the Qiskit framework, (**b**) the quantum circuit design for the $U_{\varphi }$ gate, and (**c**) the quantum circuit configuration for the *Q* gate.

The AE-QIP quantum circuit, developed using IBM Qiskit, is illustrated in Figure 3a. This circuit employs a total of five qubits, consisting of three auxiliary qubits (h = 3) and two qubits designated for data encoding. The number of classical bits (c) used for storing the measurement results is equivalent to the number of auxiliary qubits, totaling three. We assume that $|\phi_{1}\rangle$ and $|\phi_{0}\rangle$ are represented as ${\left[0.697,0.717\right]}^{T}$ and ${\left[1,0\right]}^{T}$, respectively. These states are encoded using the $U_{\phi }$ gate, as shown in Figure 3b, with corresponding RY gate rotation angles of 1.6 radians and 0 radians.

After executing the quantum circuit depicted in Figure 3a with the Qiskit simulator, a measurement of the three auxiliary qubits yielded the result “101”, which, in binary, corresponds to the decimal value y=5. Using Equation (13), the inner product $\langle \phi_{1}|\phi_{0}\rangle$ was calculated to be 0.7071, while the theoretical inner product is 0.6967. Consequently, the error rate for this result is determined to be 1.4928%, demonstrating that the AE-QIP quantum circuit is proficient in performing inner product calculations.

## 3. Algorithm Complexity

In this section, we provide a detailed definition of the error associated with the AE-QIP algorithm, denoted as ϵ. This error is intricately linked to the number of auxiliary qubits employed in the quantum amplitude estimation (QAE) algorithm. We undertake a comprehensive analysis of the AE-QIP algorithm, exploring both its space complexity and time complexity in depth. Additionally, we draw comparisons with the ST-QIP algorithm to highlight differences and similarities in their performance metrics.

### 3.1. Space Complexity Analysis

In this paper, we formalize the concept of algorithm error by defining it as ϵ and delve into the calculations of qubit consumption for the AE-QIP method. Specifically, we examine how the error level, characterized by ϵ, influences the number of auxiliary qubits needed for optimal function. We establish that when the error is maintained at ϵ, the algorithm necessitates *h* auxiliary qubits to achieve its computational objectives efficiently  [28]. Through this analysis, we aim to provide clarity on the resource requirements of the AE-QIP algorithm in the context of quantum computation.

(14) $$h=\lceil \log\frac{\pi +\sqrt{4\epsilon +1}\pi +2\epsilon }{4\epsilon^{2}}\rceil$$

During the execution of quantum inner product calculations, the total qubit consumption for the AE-QIP algorithm can be expressed as follows:

(15) $$C_{1}=\left\lceil \log\left(N\right)\right\rceil +1+h$$

where *N* denotes the dimension of the feature vector.

In contrast, for the ST-QIP algorithm, as illustrated in Figure 1, the probability of measuring the |0〉 state in the auxiliary qubits is given as follows:

(16) $$P=\frac{1}{2}\left(1+{\langle \varphi |\phi \rangle }^{2}\right)$$

To achieve the desired precision ϵ, the required number of iterations is $O\left(\frac{P(1-P)}{\epsilon^{2}}\right)$ [16], leading to the following qubits consumption:

(17) $$C_{2}=T\left(2\left\lceil \log\left(N\right)\right\rceil +1\right)$$

where $T=\frac{P(1-P)}{\epsilon^{2}}$ denotes the total number of iterations of the quantum circuit.

The space complexities of the AE-QIP and ST-QIP algorithms can be summarized as O(log(N)) and O(2Tlog(N)), respectively. Given that the probability *P* is constrained within the interval P∈(0,1), the maximum number of required iterations occurs when P=0.5 for a specified ϵ. To simplify the subsequent analysis, we assume P=0.5, which covers all potential values of *P*.

**Figure 4:**
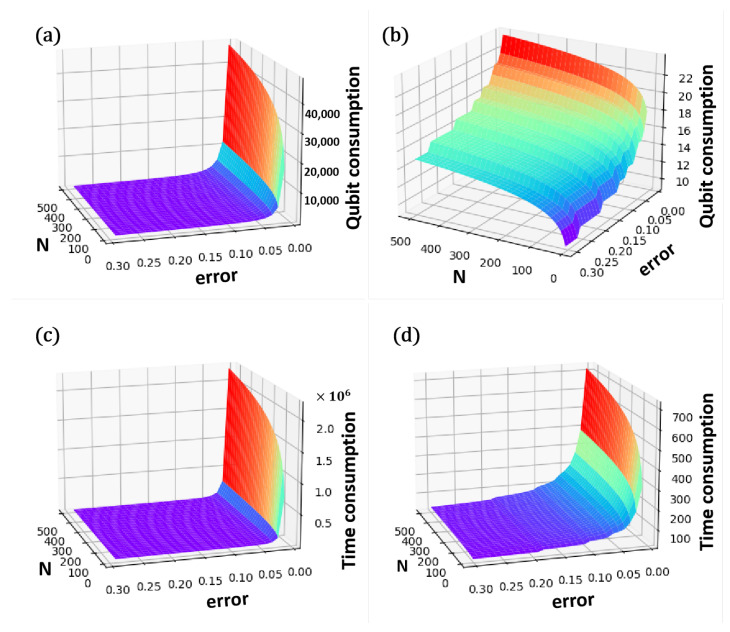
A 3D visualization depicting the space complexity, time complexity, and influencing factors of the ST-QIP and AE-QIP algorithms. (**a**) The relationship between qubit consumption, error rates, and data dimension *N* in the ST-QIP algorithm. (**b**) The relationship between qubit consumption, error rates, and data dimension *N* in the AE-QIP algorithm. (**c**) The relationship between time consumption, error rates, and data dimension *N* in the ST-QIP algorithm. (**d**) The relationship between time consumption, error rates, and data dimension *N* in the AE-QIP algorithm.

To facilitate a more intuitive comparison of the space complexity between the two algorithms, we present a three-dimensional visualization. Observations from Figure 4a,b indicate that the qubit consumption for both methodologies escalates as the error ϵ diminishes. Notably, when the error falls below a critical threshold (ϵ<0.5), the space complexity associated with the AE-QIP algorithm is shown to be superior to that of the ST-QIP algorithm. In practical applications, it is often the case that the error rate is needed to maintained below 0.05. Therefore, within an acceptable error margin, the qubit consumption of the AE-QIP algorithm is significantly lower than that of its ST-QIP counterpart.

### 3.2. Time Complexity Analysis

In the context of feature vector analysis, the execution of the AE-QIP algorithm for calculating the inner product of $L_{2}$ normalized feature vectors, as illustrated in Figure 2, demonstrates that its algorithmic complexity is predominantly derived from the quantum amplitude estimation layer. The time complexity of the AE-QIP algorithm is characterized by the expression $O(\epsilon^{-1}\log\left(N\right)+h^{2})$, where *h* is specified in Equation (14). Conversely, the ST-QIP algorithm exhibits a time complexity defined by $O(T\epsilon^{-1}\log\left(N\right))$. For the purposes of simplifying the analysis, we have adopted the assumption that P=0.5 within the ST-QIP framework.

Figure 4c,d elucidate the relationship between the time complexities of both methodologies, revealing that complexity increases with both the dimensionality of the vector *N* and the algorithm accuracy, represented as 1−ϵ. Notably, when the error term remains below a particular threshold (ϵ<0.36), the AE-QIP algorithm demonstrates a lower time complexity compared to the ST-QIP algorithm. Conversely, should the error exceed an excessive limit, the algorithm loses its practical applicability. Thus, the AE-QIP algorithm’s time complexity is significantly reduced in comparison to that of the ST-QIP algorithm when the error resides within an acceptable range, such as ϵ<0.05.

This subsection provides a comprehensive comparison of the AE-QIP algorithm and the ST-QIP algorithm, focusing on both time complexity and space complexity. As the dimensionality of the feature vector *N* expands, there is a concomitant increase in algorithmic complexity. Similarly, a decrease in the error rate ϵ corresponds to an escalation in complexity. However, when ϵ is maintained below a designated threshold, the AE-QIP algorithm exhibits a more favorable performance profile, exhibiting both lower space and time complexities relative to the ST-QIP algorithm. It is thus concluded that within the error tolerance limits applicable to practical scenarios, the AE-QIP algorithm consistently proves to be superior in terms of complexity, highlighting its significant performance advantages.

## 4. Image Retrieval Experiments

This paper addresses the challenge associated with the ST-QIP algorithm’s reliance on multiple quantum measurements, which involve wavefunction collapse and result in significant resource overhead. To mitigate this issue, we propose the AE-QIP algorithm for image similarity computations, which offers a reduction in algorithmic complexity relative to the ST-QIP approach. This section is organized into two key components. First, we examine the application of the AE-QIP algorithm in the context of image retrieval, providing a comprehensive description of how quantum inner products can be efficiently implemented using the AE-QIP quantum circuit. The effectiveness of the AE-QIP algorithm for image retrieval is subsequently validated through experimental results. The corresponding pseudo-code for the AE-QIP algorithm is provided in the Appendix A. Second, we evaluate the performance of the AE-QIP algorithm by investigating the influence of the number of auxiliary qubits on its average error rate and compare its performance with that of the ST-QIP algorithm.

### 4.1. Application of AE-QIP Algorithm in Image Retrieval

**Figure 5:**
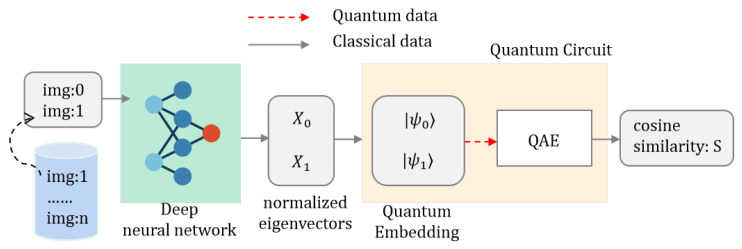
Image retrieval system process.

The primary objective of image retrieval involves comparing the feature vector of a query image with the feature vector of images in the database and measuring the similarity between them using cosine distance. This process aims to identify images whose cosine similarity *S* exceeds a defined threshold $S_{0}$. In this subsection, we illustrate the application of the AE-QIP algorithm for image retrieval and provide a schematic representation of the image retrieval system’s functioning. As depicted in Figure 5, the system comprises two main components: the classical feature vector extraction stage and the quantum state evolution stage, which together form a hybrid quantum-classical algorithm architecture.

In the classical feature vector extraction phase, the primary objective is to derive feature vectors from images to facilitate the computation of cosine similarity. We utilize a traditional neural network model for image feature vector extraction, which comprises stacked convolutional layers and pooling layers for feature map generation, followed by a fully connected network in the output layer. This layer condenses the image features into an eight-dimensional vector, which is suitable for encoding into quantum states of a specific dimension. The implementation is carried out using the PyTorch framework and trained on the ImageNet dataset, enabling us to continuously extract eight-dimensional feature vectors from images.

In this experiment, we first deploy the trained deep neural network to obtain the feature vector of the query image (denoted as img:0) and the feature vectors of the comparison images img:i, where i={1,…,m} corresponds to the i-th image retrieved from the database. The resulting feature vectors undergo $L_{2}$ normalization to yield the normalized feature vectors ${\vec{X_{0}}}^{\prime }$ and ${\vec{X_{i}}}^{\prime }$. Consequently, each image can be represented by its corresponding normalized feature vector. Specifically, Table 1 presents the query image img:0 alongside all contrast images img:i (for i=1,…,6) in the form of $L_{2}$ normalized feature vectors. In the subsequent phase of the quantum inner product algorithm, these vectors will be encoded into quantum states and processed using the quantum circuit outlined earlier.

In the subsequent analysis, the feature vectors ${\vec{X_{0}}}^{\prime }$ and ${\vec{X_{i}}}^{\prime }$ are encoded into quantum states $|\psi_{0}\rangle$ and $|\psi_{i}\rangle$ within Hilbert space via a quantum embedding layer. The quantum state inner product is then computed employing the AE-QIP algorithm, followed by a quantum measurement to ascertain the cosine similarity *S* between the feature vectors. Images are classified as similar if the cosine similarity *S* exceeds a predetermined threshold $S_{0}$, thereby facilitating the identification of analogous images.

For this experiment, we set the threshold at $S_{0}=0.9$. When the cosine similarity meets the condition $S>S_{0}$, it indicates the successful identification of a similar target. To ensure a high level of accuracy, the algorithm demands an error margin ϵ<0.05. Through Equation (14), it is determined that a minimum of seven auxiliary qubits is required for the first register. For example, let img:0 represent the query image and img:1 denote the comparison image. The theoretical assessment of the inner product of the feature vectors yields a value of 0.9809, indicating a cosine similarity of 0.9809 between the two images. The quantum state inner product is computed by executing the AE-QIP algorithm on the IBM Qiskit simulator. A single measurement suffices to derive the result, represented as ‘1000110’ in binary form, with the corresponding decimal value y=70. According to Equation (13), the inner product is calculated to be 0.9569, demonstrating that the inner product can be evaluated with an error rate of 2.4467%.

A parallel experiment was conducted with additional images within the database, successfully identifying similar images in six instances, thereby validating the efficacy of the quantum algorithm for image retrieval tasks. Table 2 delineates the experimental outcomes pertaining to image similarity computed via the AE-QIP algorithm. Within this context, “theoretical value” and “experimental value” denote the theoretically derived inner product and the experimentally obtained inner product, respectively, while “error rate” reflects the divergence between the theoretical and experimental inner products.

It is important to mention that we employed seven auxiliary qubits in our experiments, as the quantum inner product result can be represented by $\frac{2^{7}}{2}$ possible outcomes following measurement. In reality, the AE-QIP algorithm can achieve a lower error margin by increasing the number of auxiliary qubits; however, this comes at the expense of greater algorithmic complexity.

### 4.2. Performance Analysis of AE-QIP Algorithm

In Figure 4a, it is evident that the qubit consumption of the ST-QIP algorithm rises as the error decreases. This trend becomes particularly pronounced when ϵ<0.1, where we observe a sharp increase in qubit consumption with diminishing error. In this experiment, we set the dimension of the feature vector to N=8. To achieve a minimum accuracy of 95%, the error must be below 0.05, resulting in a qubit consumption of at least 700 for the ST-QIP algorithm. In contrast, the AE-QIP algorithm requires only a single measurement of the auxiliary qubits to compute the inner product. As illustrated in Figure 4b, the number of auxiliary qubits increases as the error decreases. In this experiment, four qubits are needed to encode the quantum state. According to Equation (14), when ϵ<0.05, at least seven auxiliary qubits are necessary, leading to a total qubit consumption of just 11.

To validate these theoretical calculations, we conducted simulation experiments on IBM’s Qiskit platform. To minimize experimental bias from individual test samples, we computed the average error $\hat{\epsilon }$ for the six samples in this study. Additionally, we examined how varying the number of auxiliary qubits in the AE-QIP algorithm affects the average error $\hat{\epsilon }$, as well as the impact of different measurement counts on the average error $\hat{\epsilon }$ in the ST-QIP algorithm during our experiments.

**Figure 6:**
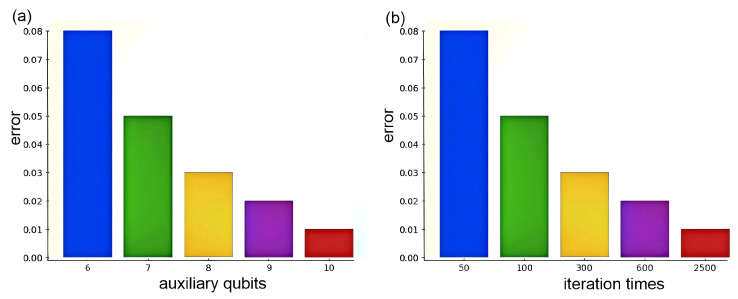
The relationship between the number of auxiliary qubits vs. algorithm error and the number of iterations vs algorithm error. (**a**) demonstrates the impact of the number of auxiliary qubits on the average error of the AE-QIP algorithm, while (**b**) highlights how varying the number of iterations affects the average error rate of the ST-QIP algorithm.

As shown in Figure 6a, increasing the number of auxiliary qubits in the first register leads to a gradual decrease in the average error of the AE-QIP algorithm. It is noteworthy that when the number of auxiliary qubits exceeds seven, the average error $\hat{\epsilon }$ remains below 0.05. At this stage, the total qubit consumption is 11, and the computational time of the algorithm is 109. In comparison, the ST-QIP algorithm requires multiple measurements to achieve equivalent accuracy. Figure 6b shows that when the number of iterations exceeds 100, the average error converges to $\hat{\epsilon }<0.05$. Thus, while the AE-QIP algorithm achieves an accuracy comparable to that of the ST-QIP algorithm using only 11 qubits, the latter requires 700 qubits consumption and incurs substantially higher computational time. This highlights that the algorithmic complexity of AE-QIP is considerably lower than that of ST-QIP. With a potential for higher accuracy requirements, the advantages of the AE-QIP algorithm become increasingly evident. Therefore, the AE-QIP algorithm outperforms the ST-QIP algorithm when the required accuracy for practical applications is below a certain threshold, such as ϵ<0.05.

## 5. Conclusions and Discussion

The most critical aspect of an image retrieval system is calculating image similarity, which can be quantified using the cosine distance between feature vectors. In this paper, we theoretically establish the connection between quantum and classical algorithms for cosine similarity, providing a foundation for implementing a quantum similarity algorithm. Additionally, while the swap test has traditionally played a pivotal role in quantum inner product applications in previous quantum similarity algorithms, this measurement method is both resource-intensive and time-consuming. To address this, we introduce a quantum inner product algorithm based on amplitude estimation. By employing quantum amplitude estimation instead of swap tests, we enhance the quantum inner product algorithm without necessitating multiple executions of quantum circuits. Our complexity analysis demonstrates that the time and space complexities of the AE-QIP algorithm are significantly reduced compared to those of the ST-QIP algorithm. Furthermore, simulation results of image similarity calculations indicate that the AE-QIP algorithm can achieve the same level of accuracy as the ST-QIP algorithm while utilizing far fewer qubits. This highlights the superior overall performance of the AE-QIP algorithm, making it well-suited for image similarity calculations within image retrieval systems.

This paper focuses specifically on the quantum algorithm component of image similarity within the broader context of quantum image retrieval systems. However, there is still significant work ahead. For example, replacing classical deep neural networks with quantum neural networks will allow for the direct extraction of the quantum state feature vector from images, paving the way for a comprehensive quantum state retrieval system. Furthermore, in the practical application of image similarity computation, it is often essential to utilize high-dimensional feature vectors to preserve the accuracy of the algorithm. However, quantum computers encounter significant challenges when processing high-dimensional data. These challenges can be mitigated through the application of principal component analysis (PCA), which reduces the dimensionality of the data while maintaining their essential characteristics. Unfortunately, the classical PCA algorithm exhibits high computational complexity, on the order of $O(N^{2})$. Overcoming these challenges through efficient quantum algorithms, while achieving overall improvements in the performance of quantum retrieval systems, will constitute a key focus of our future research endeavors.   

## Figures and Tables

**Table 1:** The $L_{2}$ normalized feature vectors.

Type	Image	Feature Vector
Query image	img.0	[0.325,0.102,0.280,0.843,0.160,0.133,0.125,0.189]
Comparison images	img.1	[0.215,0.004,0.339,0.857,0.061,0.170,0.150,0.223]
	img.2	[0.829,0.153,0.279,0.161,0.065,0.041,0.151,0.397]
	img.3	[0.907,0.160,0.046,0.052,0.130,0.202,0.084,0.289]
	img.4	[0.115,0.032,0.355,0.068,0.386,0.391,0.622,0.408]
	img.5	[0.178,0.345,0.322,0.114,0.015,0.004,0.786,0.339]
	img.6	[0.061,0.081,0.215,0.028,0.777,0.481,0.059,0.322]

**Table 2:** The cosine similarity and error rate were computed using the AE-QIP algorithm.

No.	y Value	Experimental Value	Theoretical Value	Error Rate
1	70	0.9569	0.9809	2.4467%
2	83	0.5957	0.6086	2.1196%
3	86	0.4714	0.4806	1.9143%
4	86	0.4714	0.466	1.1588%
5	87	0.4276	0.4446	3.8237%
6	88	0.3827	0.3684	3.8817%

## Data Availability

The original data and Code presented in this study are openly available at https://github.com/RichardYang88/Q_img_retrieval (accessed on 17 December 2024).

## Appendix A. Pseudo-Code for the AE-QIP Algorithm

**Algorithm A1** Main Routine
1: **for** i=0 to 5 **do** 2: Compute $\theta_{i},\beta_{i}$ using normalized x1,x2 3: Run AE-QIP circuit with parameters $\theta_{i},\beta_{i}$ 4: Get quantum counts 5: Compute $q\_out=-\cos(\pi \cdot y/2^{m-1})$ with m=7 6: Compute c_out=x1·x2 7: Compute error rate: $err=\frac{\left|q\_out-c\_out\right|}{c\_out}$ 8: Display results 9: **end for**

**Algorithm A2** AE-QIP Circuit
1: Initialize circuit with *n* qubits 2: Apply Hadamard on m auxiliary qubits 3: Apply *U* layers with control and inverse transformations 4: **for** each qubit in auxiliary qubits **do** 5: Apply controlled *Q* layers 6: **end for** 7: Apply $QFT^{†}$ gate on auxiliary qubits 8: Measure qubits and return counts
